# Determinants of HIV Testing Among Migrant Men Who Have Sex With Men from Sub-Saharan Africa and Other Regions Residing in 10 European Countries

**DOI:** 10.1007/s10461-023-04239-1

**Published:** 2024-02-08

**Authors:** Oladipupo Shobowale, Axel J. Schmidt, Paula Meireles, Daniela Rojas Castro, Sandrine Detandt, Sarah E. Stutterheim, Peter Weatherburn, Kai J. Jonas

**Affiliations:** 1 Department of Work and Social Psychology, Maastricht University, Universiteitssingel 40, 6229 ER Maastricht, The Netherlands; 2 London School of Hygiene & Tropical Medicine (LSHTM), Sigma Research, London, UK; 3 EPIUnit - Instituto de Saúde Pública, Universidade do Porto, Porto, Portugal; 4 Coalition PLUS, Community-Based Research Laboratory, Pantin, France; 5 Faculty of Psychology, Observatoire du Sida et des Sexualités Research Center, Université Libre de Bruxelles (ULB), Brussels, Belgium; 6 Department of Health Promotion, Care and Public Health Research Institute, Maastricht University, Maastricht, The Netherlands; 7 Laboratório para a Investigação Integrativa e Translacional em Saúde Populacional (ITR), Porto, Portugal

**Keywords:** HIV/AIDS, HIV testing, Migrant men who have sex with men, Europe

## Abstract

Migrant men who have sex with men (mMSM) from sub-Saharan Africa (SSA) and other regions outside Europe are highly vulnerable to HIV. However, research on the determinants of HIV testing among mMSM from SSA, and how these differ across the categories of mMSM living in Europe, is limited. Using data from the European MSM Internet Survey (EMIS-2017), we assessed HIV testing prevalence and recency in mMSM from SSA and other mMSM residing in ten European countries, as well as the determinants of HIV testing across different mMSM categories with logistic regression analyses. Ever-testing for HIV was slightly higher in mMSM from SSA (83%) compared to other mMSM categories (75–80%), except for mMSM from Latin America and Caribbean region (84%). Overall, 20% of mMSM had never tested. In multivariable analysis, higher age (adjusted odds ratio [AOR] 1.05, 95% confidence interval [CI] 1.01–1.10), higher HIV knowledge (AOR 1.45, 95%-CI 1.11–1.90), and residence in smaller settlements (AOR 0.45, 95%-CI 0.21–0.96) were significantly associated with ever testing for HIV in mMSM from SSA. Comparing mMSM from SSA to mMSM from other regions, we found varying significant similarities (higher age, residence in smaller settlements and HIV knowledge) and differences (lower educational attainment, not identifying as gay, being a student, and limited disclosure of homosexual attraction) in the determinants of ever-testing for HIV. Community-specific interventions addressing identified sociodemographic and behavioral determinants to increase HIV testing uptake in the different mMSM categories and better data for further research are warranted.

## Introduction

The HIV epidemic remains a significant global health problem. In 2014, the Joint United Nations Program on HIV and AIDS (UNAIDS) set the 90–90–90 targets as part of the Fast-Track strategy towards ending the epidemic by 2030; with 90% of people living with HIV knowing their status, 90% of those aware of their status on antiretroviral therapy (ART), and 90% of people on ART achieving viral suppression [1]. While the vast majority of European Economic Area (EEA) countries, as well as the United Kingdom (UK) and Switzerland, have made commendable progress in achieving these targets, specific subpopulations within these regions still lag behind in access to testing and treatment services [2].

According to the European HIV/AIDS surveillance data from 2021, men who have sex with men (MSM) and migrants accounted for, respectively, 40.0% and 42.0% of new HIV diagnoses in the EEA [3], with migrants having a greater likelihood of delayed diagnosis and late presentation to care [3–5]. Migrant men who have sex with men (i.e., defined here as migration from outside Europe into the EEA, the UK and Switzerland, and hereafter referred to as migrant MSM [mMSM]) are particularly vulnerable. In fact, the 2021 regional surveillance data indicated that, although new HIV diagnoses in non-migrant MSM have steadily declined over the past ten years, there has been hardly any decline in new HIV diagnoses among mMSM [3]. Migrant MSM are at higher risk for HIV acquisition before, during, and after migration [6–8]. Especially in post-migration contexts, acquisition estimates were as high as 72% among mMSM [9]. Additionally, recent evidence suggests that disparities in HIV status awareness persist among mMSM in some European countries. In a Belgian study, Marty et al. [10], for example, reported a higher prevalence of undiagnosed HIV among mMSM with non-European nationality (534.2 per 10,000) compared to MSM of Belgian nationality (55.6 per 10,000), and other MSM with European nationality (151.3 per 10,000). Similar findings on the higher proportion of undiagnosed HIV among mMSM have also been reported in Spain [11] and France [12].

The role of HIV testing as a gateway to HIV treatment and prevention cannot be overemphasized. It enhances linkage to care, prompts initiation of ART for the treatment and prevention of further HIV transmission [13, 14], and promotes individual behavior modification in those aware of their HIV status [15, 16]. Nonetheless, knowledge of HIV infection remains the weakest link in the HIV care continuum [17], and notably in countries with generalized epidemics [18, 19]. Migration to Europe and other global North countries from these countries will continue to rise into the foreseeable future [20, 21], and this calls for improved access to HIV testing to mitigate health inequalities related to late HIV diagnosis among mMSM. Furthermore, as countries embark on attaining the ambitious UNAIDS 95–95–95 targets by 2025 [2], adequate engagement of migrants from specific regions in the HIV care continuum, which starts with HIV testing, is critical to reaching these targets and ending the HIV epidemic.

There is still a dearth of research on HIV testing among mMSM from sub-Saharan Africa (SSA) in Europe [22], with most studies originating from the UK [23], often with small sample sizes: sample size (*n*) ranged between 7 and 76 [24–28], even using aggregated samples of “Black” MSM [25, 29]. Furthermore, HIV testing studies from Europe investigating the intersection between sexual identity, ethnicity and/or migration [30], including migration generation status are lacking. Studies on migrants from SSA done in Germany [31, 32], Portugal [33, 34], France [35, 36], and Belgium [37, 38] have largely focused on heterosexual men and women. This is further corroborated by findings from two systematic reviews [39, 40] showing that most studies conducted in high-income countries on the determinants of HIV testing among people with a migration background have focused on heterosexual individuals and specific subgroups. In sum, there is limited information coming from European countries on mMSM from SSA and other groups of mMSM. Thus, it is important to close these evidence gaps.

Existing studies including mMSM in their analyses have investigated migration background (rather broadly defined) as one of the determinants of HIV testing [29, 41–43]. However, the use of broad categories, such as foreign vs. non-foreign born [41], white vs. non-white ethnicity [44], Western and non-Western migrant [45], and country or region of birth [42, 43, 46], in determining migration status may be too limited. An improved investigation of migration status, in all of its complexity, may be needed to identify differences that exist between these categories. As an example, an individual may have been born in a foreign country to expatriate parents, but not necessarily identify with the country of birth. Such an individual may be classified as a “migrant” due to their place of birth, but, in fact, that person is not very similar in HIV testing behavior to individuals originating from the same country. Such individuals may fit more with the mainstream culture of the country of residence or “destination country”.

Groups of people with a migration background are heterogeneous and there may be differences in the determinants of HIV testing [40]. Thus, it is essential to investigate the differential influences of these factors on HIV testing in different migrant categories. For example, Kuehne and colleagues [32], in a study conducted among migrants from SSA in Germany, found that HIV testing was associated with higher educational attainment, greater HIV knowledge, a previous STI diagnosis, less HIV stigma, and more discussions about HIV within the community. Younger age, having varied sexual partners, and recent migration was associated with lesser likelihood of testing in current country of residence. Ojikutu et al. [47], also established differences in the determinants of testing among U.S.-born and non-U.S.-born Black individuals with Sub-Saharan Africa, Caribbean, and Haitian backgrounds. Older age and increased length of residence was associated with lesser likelihood of testing. Ojikutu and colleagues [47] also reported higher HIV knowledge and lower HIV stigma index scores in SSA migrants compared to Caribbean migrants. Differences in the effect of migration origin on HIV testing have also been reported in other studies [48–50]. This warrants approaches that attempt to adequately classify individuals with a migration background into appropriate categories such as the use of self-identified ethnic or racial background in addition to previously highlighted proxies such as country of birth. Furthermore, it is important to identify profiles of mMSM within these categories who have never tested for HIV to develop relevant strategies for HIV testing and sexual health promotion.

In this analysis, we set out to (1) investigate HIV testing prevalence and recency among mMSM from SSA and other mMSM of non-European origin, including mMSM with Eastern European origin, and (2) ascertain how determinants of HIV testing differ between mMSM from SSA and mMSM from other regions. Building on prior research, we focused our analyses on examining the association between HIV testing and sociodemographic and sexual behavioral characteristics used in these studies [8, 29, 41–45, 51–53].

## Methods

### Study Design and Population

We used data from the European MSM Internet Survey (EMIS-2017) which was an anonymous, online, cross-sectional survey available in 33 languages and completed by MSM in 50 countries, including some non-European countries (Canada, Israel, Lebanon and the Philippines) between October 2017 and January 2018 (www.emis-project.eu). Full details on methods of EMIS including eligibility criteria are described elsewhere [54, 55].

Given our interest in mMSM from SSA and other regions, for this analysis, our inclusion criteria included participants indicating country of birth from and/or of SSA, Latin America and Caribbean, Middle East and North African, South-East Asian, Eastern European and Western Pacific origin living in the following ten European countries: Belgium, France, Germany, Greece, Italy, Netherlands, Portugal, Spain, Switzerland, and the United Kingdom (see *Migrant Background Categorization* paragraph below for full details). The ten countries of residence were selected based on sufficiently large numbers of mMSM from SSA, our primary group of interest, participating in the survey. See Annex (Table 4) for details.

### Migration Background Categorization

Focusing on non-European and Eastern European migrants residing in the ten countries selected, and to assign mMSM into a migrant category, we used the four questionnaire items: country of birth, country of residence (“*Were you born in country of residence or another country?”*); ethnic or racial minority membership (“*Do you consider yourself a member of an ethnic or racial minority?”);* description of minority group membership (“*What minority group are you a member of?”*).

In the first step, men born outside their country of residence who answered additional questions on ethnic or racial minority membership (open write-in field for ethnic or racial minority group) were assigned to the appropriate migrant category and as first generation mMSM. For example, migrant category assignment was ascertained with participants response on the open write-in field for ethnic minority status and group membership e.g., “Turkish”, “African”, “Arab”, “Chinese”, in order to include non-primary migration backgrounds. Participants were assigned into different migrant background categories under the following World Health Organization (WHO) regional categories: “sub-Saharan Africa” (SSA), “Latin America and Caribbean” (LA&C), “Middle East and North Africa” (MENA), “South-East Asia Region” (SEAR), “Eastern Europe (EE, *i.e.,* former Soviet Union republics including Baltic states)” and “Western Pacific Region excluding Australia and New Zealand” (WPR). See Annex (Table 5) for full details of participants’ country of origin.

In the next step, men born in their country of residence who answered additional questions on ethnic or racial minority membership were assigned to the appropriate migrant category, and also coded as second/other generation mMSM. For first and second/other generation mMSM, participants reporting multiple ethnic or racial background (e.g., mixed ethnicity) were assigned to the self-identified minority group when this information was available.

Migrants from Australia, New Zealand, the United States of America, Canada, and European Economic Area (EEA) countries, the UK and Switzerland were excluded from our analysis because of predominant ethnic and cultural similarities with European MSM in the ten countries selected for this analysis [56], unless information on ethnic-racial minority group membership was available. We also excluded participants with vague, unclear, or incomplete information on their ethnic or racial background (e.g., simply stating Black, as this did not allow categorization as SSA or LA&C origin) from our analysis. Further details on migrant categories and country distributions of our analytic sample are shown in Annex (Table 6).

After assigning individuals to the categories reflecting migration background, 7303 mMSM were retained in the analysis. Overall, 13.4% (n = 981) of mMSM were living with diagnosed HIV, and subsequently excluded from our analysis. This resulted in a final sample size of 6322 untested and HIV negative mMSM for the analysis.

### Measures

*HIV testing status* was measured with the question, “Have you ever received an HIV test?” with responses categorized as “Yes” or “No”. A response of yes (in effect, ever having tested for HIV) was used as the primary outcome measure for our analysis.

*HIV testing recency* was measured with the question, “When did you last go for an HIV test?” with responses: “Within the last 24 h”; “Within the last 7 days”; “Within the last 4 weeks”; “Within the last 6 months”; “Within the last 12 months”; “Within the last 5 years”; “More than 5 years ago”, and “Never”. This was collapsed into “within previous 12 months”, “more than 12 months” and, “Never tested”.

#### Sociodemographic characteristics

Age was ascertained with the question, “How old are you?”. Participants’ responses in years were used as a continuous variable.

Migrant generation status was ascertained during migration background categorization with the question, “Were you born in country of residence or another country?”. This was categorized as “First generation” and “Second/other generation”.

Educational attainment was ascertained with the question, “How many years have you spent in full-time education since the age of 16?”. This was reported in years and then categorized into a dichotomous variable (“Low to moderate” and “High”), based on the ISCED-1997 classification, with 6 years or more spent in full-time education since the age of 16 broadly classified as tertiary education and above (“High”).

Settlement size was ascertained with the question, “How would you describe the place you live in?” with responses: “A very big city or town (a million or more people)”; “A big city or town (500,000–999,999 people)”; “A medium-sized city or town (100,000–499,999 people)”; “A small city or town (10,000–99,999 people)”; “A village/the countryside (less than 10,000 people)”. This was collapsed into a dichotomous variable (large cities/town “more than 500,000 people” and small to medium cities/town “less than 500,000 people”).

Sexual identity was ascertained with the question, “Which of the following options best describes how you think of yourself?” with responses: “Gay or homosexual”; “Bisexual”; “Straight or heterosexual”; “Any other term”; “I don’t usually use a term”. This was categorized as “Gay or homosexual” and responses other than “Gay or homosexual” were categorized as “Not gay or homosexual”.

Occupation was ascertained with the question, “Which of the following best describes your current occupation?” with responses: “Employed full-time”; “Employed part-time”; “Self-employed”; “Unemployed”; “Student”; “Retired”; “Long-term sick leave/medically retired”; “Other”. This was collapsed into “Employed”, “Unemployed”, “Student”, and “Other (retired/long term sick leave/medically retired and other)”.

Financial status/coping was ascertained with the question, “Which of these phrases would you say comes closest to your feelings about your income these days?” with the following responses: “Living really comfortably on present income”; “Living comfortably on present income”; “Neither comfortable nor struggling on present income”; “Struggling on present income”; “Really struggling on present income”. This was categorized as “Living really comfortably/comfortably”, “Neutral”, and “Really struggling/struggling”.

#### Disclosure of Homosexual Attraction (Outness)

Men who were attracted to men were asked about their disclosure of homosexual attraction (outness). This was ascertained with the question, “Thinking about all the people who know you (including family, friends and work or study colleagues), what proportion know that you are attracted to men?” with the following response options: “None”; “Few”; “Less than half”; “More than half”; “All or almost all”. This was collapsed into a dichotomous variable (“Out to all/more than half of family and friends or colleagues” and “Out to few/less than half of family, friends or colleagues or not out”).

#### HIV Testing, Treatment and Transmission Knowledge

Knowledge related to HIV was assessed from 10 statements on HIV testing, treatment and transmission measured with a five-point knowledge response set: “I knew this already”; “I wasn’t sure about this”; “I didn’t know this already”; “I don’t understand this”; “I do not believe this”. These were “AIDS is caused by a virus called HIV”; “You cannot be confident about whether someone has HIV or not from their appearance”; “There is a medical test that can show whether or not you have HIV”; “If someone becomes infected with HIV, it may take several weeks before it can be detected in test”; “There is currently no cure for HIV infection”; “HIV infection can be controlled with medicines so that its impact on health is much less”; “A person with HIV who is on effective treatment (called ‘undetectable viral load’) cannot pass their virus to someone else during sex”; “HIV cannot be passed during kissing, including deep kissing, because saliva does not transmit HIV”; “You can pick up HIV through your penis while being 'active' in anal or vaginal sex (fucking) without a condom, even if you don’t ejaculate”; “You can pick up HIV through your rectum or vagina while being 'passive' during sex (being fucked).” We computed HIV knowledge by recoding each of the ten statements into a dummy variable with value 1 assigned to those answering, “I knew this already” and value 0 for all other answers/responses. We subsequently developed an additive scale with all the 10 statements ranging from 0 to 10. Higher scores on this variable indicated higher knowledge. Extensive details on the questionnaire and response options are described elsewhere [55].

#### Sexual Behavioral Characteristics

The number of male sexual partners in the previous 12 months was grouped into “0”, “1–10”, and “more than 10 partners”. Condomless anal intercourse (CAI) in the previous 12 months with any sex partner was recoded and categorized as a dichotomous variable, “Yes” or “No”. Those without male sexual partners in the previous 12 months were categorized as “No”.

### Data Analyses

Descriptive statistics were computed for all variables included in our analysis using frequencies for categorical variables and means with standard deviations (SDs) for continuous variables. Comparisons between groups were conducted using χ2 tests for categorical variables and the Kruskal–Wallis test for continuous variables (assumptions of normality were violated). Univariate logistic regression analyses were conducted to assess the associations between our primary study outcome measure (ever testing for HIV) and each predictor outlined above for the different migrant categories. Odds ratio (OR) and 95% confidence intervals (CIs) were calculated. Subsequently, we built multivariable logistic regression models using the same predictors for each migrant category. Adjusted odds ratio (aOR) with 95%-CI were calculated to identify predictor variables that were associated with our study outcome in the multivariable models. In the multivariable analysis, a *p*-value < 0.05 was considered statistically significant. Furthermore, we used Firth’s logistic regression (penalized likelihood) for migrant categories with relatively small sample sizes to reduce the chances of unreliable and biased coefficients resulting from the use of Maximum Likelihood Estimation logistic regression-based methods [57] using the STATA “firthlogit” command. This approach has been reported to produce estimates that are more accurate than maximum likelihood logit and probit estimates [58, 59]. The fit of the final logistic regression model was assessed using the Hosmer–Lemeshow goodness of fit test when the likelihood ratio test was employed for categories with large sample sizes. The Cragg-Uhler/Nagelkerke *R*^*2*^ was used for the penalized logistic regression analysis. We also assessed variables in each migrant category (model) for multicollinearity. Tolerance coefficients ranged between 0.201 and 0.994 across the categories. Variance inflation factors ranged between 1.02 and 4.99 across categories. Hence, no predictor variables were removed from our models. Missing data ranged between 0 and 6.8% across predictors and was handled as completely at random. Sensitivity analyses excluding participants with discrepant data (multi-inconsistent responses) did not appreciably change the results of logistic regression models across all mMSM categories, with results remaining largely the same as with the total sample of participants (see Annex, Table 7). To avoid an appreciable decrease in sample sizes, results from the total sample were used in this paper. All statistical analyses were performed using STATA version 16 [60].

## Results

### Participant Characteristics

After assignment to the different migrant categories, mMSM from SSA, LA&C, MENA, SEAR, EE and WPR respectively accounted for 4.9%, 34.5%, 17.8%, 7.3%, 29.6% and 5.9% of the study sample. The mean age of mMSM included in the analyses was 33.4 (SD:10.7). The majority of mMSM were first-generation migrants (84.8%), identified as gay or homosexual (76.7%), were employed (65.5%), and had high educational attainment (67.6%). More than half (55.1%) lived in big cities, and 21.9% were students. Furthermore, 19.2% of mMSM in our study were struggling financially. About 46.0% of mMSM had not fully disclosed their homosexual attraction to others. There was a high level of knowledge of HIV (mean score of 8.8 [SD:1.6]).

Overall, all variables included in this analysis—except for educational attainment—were found to differ significantly across migrant categories. Table 1 shows the descriptive statistics for all the variables analyzed for this paper.

**Table 1:** Study sample characteristics of mMSM (all mMSM categories and included variables)

	Total	SSA	LA&C	MENA	SEAR	EE	WPR	χ^2^/*H*
N	6322	311	2181	1125	458	1872	375	
Age								
Age*	33.36 ± 10.7	35.30 ± 12.5	34.04 ± 11.1	32.53 ± 10.4	32.73 ± 11.2	33.05 ± 10.0	32.60 ± 9.84	***H*(5) = 26.69, *p* < 0.001**
Migrant generation status								
1st	5360 (84.8)	235 (75.6)	1947 (89.3)	786 (69.9)	221 (48.3)	1850 (98.8)	321 (85.6)	χ^2^ (5) = 1000, *p* < 0.001
2nd/other	962 (15.2)	76 (24.4)	234 (10.7)	339 (30.1)	237 (51.8)	22 (1.2)	54 (14.4)	
Educational attainment								
Low/Moderate	1910 (32.4)	96 (34.3)	657 (31.8)	331 (31.9)	136 (32.2)	593 (34.3)	97 (27.2)	χ^2^ (5) = 8.08, *p* = 0.152
High	3977 (67.6)	184 (65.7)	1407 (68.2)	706 (68.1)	286 (67.8)	1135 (65.7)	259 (72.8)	
Settlement size: size of city/town of residence								
500,000 or more	3443 (55.1)	165 (53.7)	1277 (59.3)	617 (55.6)	238 (52.7)	953 (51.4)	193 (51.6)	χ^2^ (5) = 28.75, *p* < 0.001
Less than 500,000	2809 (44.9)	142 (46.3)	878 (40.7)	492 (44.4)	214 (47.3)	902 (48.6)	181 (48.4)	
Sexual identity								
Gay/Homosexual	4840 (76.7)	201 (68.8)	1693 (77.8)	819 (72.9)	343 (74.9)	1468 (78.5)	316 (84.3)	χ^2^ (5) = 51.22, *p* < 0.001
Not gay/homosexual	1471 (23.3)	109 (35.2)	483 (22.2)	304 (27.1)	115 (25.1)	401 (21.5)	59 (15.7)	
Occupation								
Employed	4126 (65.5)	185 (59.7)	1366 (62.8)	686 (61.0)	295 (64.6)	1355 (72.9)	239 (63.9)	χ^2^ (15) = 105.38, *p* < 0.001
Unemployed	485 (7.7)	23 (7.4)	226 (10.4)	96 (8.5)	26 (5.7)	94 (5.1)	20 (5.4)	
Student	1382 (21.9)	76 (24.5)	483 (22.2)	278 (24.7)	113 (24.7)	330 (17.7)	102 (27.3)	
Other	306 (4.9)	26 (8.4)	99 (4.6)	64 (5.7)	23 (5.0)	81 (4.3)	13 (3.4)	
Financial status/coping								
(Really) comfortable	2833 (45.0)	122 (39.6)	850 (39.1)	517 (46.1)	219 (47.9)	931 (50.2)	194 (52.0)	χ^2^ (10) = 130.95, *p* < 0.001
Neutral	2250 (35.8)	101 (32.8)	864 (39.7)	336 (29.9)	148 (32.4)	688 (37.1)	113 (30.3)	
(Really) struggling	1207 (19.2)	85 (27.6)	460 (21.2)	269 (24.0)	90 (19.7)	237 (12.7)	66 (17.7)	
Disclosure of homosexual attraction (outness)								
Out to all/ > half	3358 (54.3)	141 (46.8)	1393 (64.5)	439 (39.7)	229 (50.4)	1007 (54.6)	176 (47.4)	χ^2^ (5) = 202.06, *p* < 0.001
Out to < half/Few/None	2851 (45.7)	160 (53.2)	767 (35.5)	667 (60.3)	225 (49.6)	837 (45.4)	195 (52.6)	
HIV testing, treatment and transmission knowledge								
HIV knowledge score*	8.80 ± 1.6	9.00 ± 1.3	8.89 ± 1.4	8.52 ± 1.9	8.84 ± 1.4	8.83 ± 1.5	8.79 ± 1.6	***H* (5) = 26.14, *p* < 0.001**
Number of male sexual partners (previous 12 months)								
0	663 (10.5)	27 (8.7)	213 (9.8)	120 (10.7)	58 (12.7)	201 (10.7)	44 (11.7)	χ^2^ (10) = 22.91, *p* < 0.05
1–10 partners	3567 (56.5)	177 (57.1)	1186 (54.4)	618 (55.0)	261 (57.1)	1105 (59.2)	220 (58.7)	
> 10 partners	2084 (33.0)	106 (34.2)	781 (35.8)	386 (34.3)	138 (30.2)	502 (30.1)	111 (29.6)	
CAI (previous 12 months)								
Yes	3841 (63.4)	201 (67.4)	1381 (66.2)	684 (63.7)	257 (57.8)	1091 (60.9)	227 (62.2)	χ^2^ (5) = 20.62, *p* = 0.001
No	2217 (36.6)	97 (32.6)	704 (33.8)	389 (36.3)	188 (42.2)	701 (39.1)	138 (37.8)	

*Mean ± SD; In Bold: Kruskal–Wallis test

*CAI* condomless anal intercourse; *SSA*, Sub-Sahara Africa; *LA&C* Latin America and Caribbean; *MENA* Middle East and North Africa; *SEAR* South-East Asia Region; *EE* Eastern Europe (i.e., former Soviet Union republics including Baltic states); *WPR* Western Pacific Region excluding Australia and New Zealand

### HIV Testing Experience

**Fig. 1:**
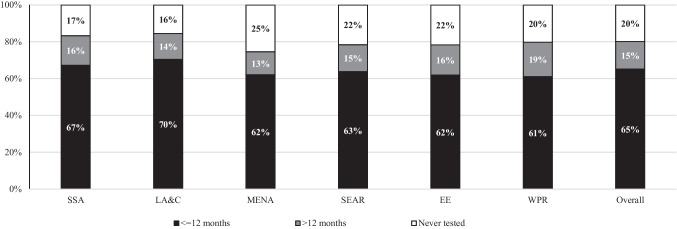
HIV testing history among migrant MSM from different world regions. *SSA* sub-Sahara Africa; *LA&C* Latin America and Caribbean; *MENA* Middle East and North Africa; *SEAR* South-East Asia Region; *EE* Eastern Europe (i.e., former Soviet Union republics including Baltic states); *WPR* Western Pacific Region excluding Australia and New Zealand; Overall: Total (aggregated) group of mMSM in study sample

Overall, 20.0% of mMSM in our final analytic sample had never tested for HIV (N = 1258). Figure 1 presents the HIV testing prevalence in the different mMSM categories. Migrant MSM with a MENA background accounted for the highest proportion of those who had never tested for HIV (25.0%). The lowest proportions of those who had never tested for HIV were observed in mMSM from LA&C (16.0%) and SSA (17.0%). The median age of never tested mMSM was 26 years (range 14–87).

### HIV Testing Recency

Overall, 35.0% of all mMSM included in our study had not tested in the 12 months prior to the survey completion. Figure 1 shows HIV testing recency in the different mMSM categories. HIV testing within the previous 12 months was reported by 65.0% of mMSM while 15.0% of mMSM reported testing longer than 12 months before survey completion.

### Determinants of HIV Testing

Table 2 presents the univariate logistic regression analyses for predicting ever testing for HIV. Across all mMSM categories, higher age was associated with higher odds of ever testing for HIV, while other sociodemographic characteristics such as low/moderate educational attainment, and being a student presented with lower odds of ever testing for HIV. mMSM living in smaller settlements and not self-identifying as gay had lesser odds of ever testing for HIV across all migrant categories except for mMSM from SSA. Migrant generation status (second/other generation) was only a significant predictor of a lesser likelihood of ever testing for HIV in mMSM from SSA. Similarly, unemployment was associated with a lesser likelihood of testing only in mMSM originating from MENA. Migrant MSM from MENA, EE and WPR who were struggling financially had a lesser likelihood of ever testing for HIV. Not fully disclosing homosexual attraction to others was associated with a lesser likelihood of ever testing for HIV in all migrant categories except for mMSM from SSA. Higher HIV knowledge was significantly associated with increased odds of ever testing across all mMSM categories. Sexual behavioral factors such as having more male sexual partners and CAI in the previous 12 months were also significantly associated with increased odds of ever being tested for HIV across all migrant categories.

**Table 2:** Univariate logistic regression analysis (OR and 95% CI) for predicting ever testing for HIV among mMSM

	SSA	LA&C	MENA	SEAR	EE	WPR
Age						
Age	1.055*** [1.023,1.087]	1.074*** [1.059,1.089]	1.062*** [1.045,1.080]	1.058*** [1.030,1.088]	1.061*** [1.047,1.076]	1.058*** [1.025,1.092]
Migrant generation status						
1st	Ref	Ref	Ref	Ref	Ref	Ref
2nd/Other	0.398** [0.213,0.742]	0.742 [0.525,1.049]	1.096 [0.816,1.471]	1.065 [0.684,1.660]	2.293 [0.614,8.564]	1.103 [0.535,2.277]
Educational attainment						
Low/Med. Educ	0.452* [0.242,0.844]	0.395*** [0.309,0.506]	0.469*** [0.351,0.627]	0.611* [0.375,0.995]	0.427*** [0.337,0.541]	0.450** [0.260,0.780]
High Educ	Ref	Ref	Ref	Ref	Ref	Ref
Settlement Size: size of city/town of residence						
> 500,000	Ref	Ref	Ref	Ref	Ref	Ref
< 500,000	0.578 [0.318,1.052]	0.417*** [0.330,0.529]	0.465*** [0.353,0.611]	0.542** [0.344,0.856]	0.448*** [0.356,0.562]	0.560* [0.335,0.934]
Sexual identity						
Gay/Homosexual	Ref	Ref	Ref	Ref	Ref	Ref
Not gay/homosexual	0.573 [0.315,1.043]	0.368*** [0.288,0.472]	0.595*** [0.445,0.795]	0.493** [0.306,0.795]	0.388*** [0.304,0.495]	0.460* [0.250,0.848]
Occupation						
Employed	Ref	Ref	Ref	Ref	Ref	Ref
Unemployed	0.596 [0.195,1.820]	0.744 [0.489,1.131]	0.337*** [0.216,0.525]	3.751 [0.704,19.985]	0.848 [0.505,1.421]	0.811 [0.243,2.705]
Student	0.333** [0.172,0.645]	0.249*** [0.192,0.322]	0.442*** [0.324,0.603]	0.385*** [0.238,0.625]	0.393*** [0.301,0.511]	0.296*** [0.172,0.509]
Other	0.688 [0.228,2.075]	0.681 [0.380,1.220]	0.770 [0.422,1.405]	1.292 [0.400,4.170]	0.616 [0.369,1.027]	0.487 [0.138,1.722]
Financial status/coping						
(Really) comfortable	Ref	Ref	Ref	Ref	Ref	Ref
Neutral	0.910 [0.449,1.844]	0.677** [0.520,0 .882]	0.596** [0.434,0.819]	0.642 [0.392,1.049]	0.676** [0.532,0.860]	0.844 [0.466,1.526]
(Really) Struggling	0.794 [0.385,1.635]	0.849 [0.615,1.174]	0.558** [0.399,0.781]	1.081 [0.576,2.029]	0.648* [0.463,0.906]	0.505* [0.265,0.963]
Disclosure of homosexual attraction (outness)						
Out to all/ > half	Ref	Ref	Ref	Ref	Ref	Ref
Out to < half/Few/None	0.654 [0.353,1.212]	0.353*** [0.279,0.448]	0.442*** [0.328,0.596]	0.375*** [0.232,0.604]	0.365*** [0.290,0.460]	0.332*** [0.190,0.578]
HIV testing, treatment and transmission knowledge						
HIV Knowledge	1.412** [1.152,1.730]	1.384*** [1.288,1.487]	1.392*** [1.293,1.498]	1.393*** [1.201,1.616]	1.458*** [1.358,1.566]	1.497*** [1.286,1.743]
Number of male sexual partners (previous 12 months)						
0	Ref	Ref	Ref	Ref	Ref	Ref
1–10 partners	1.952 [0.801,4.756]	1.787** [1.282,2.491]	1.810** [1.216,2.694]	2.769** [1.530,5.011]	2.868*** [2.105,3.907]	3.056** [1.566,5.965]
> 10 partners	3.295*	4.384***	5.949***	5.285***	8.731***	9.028***
	[1.214,8.944]	[2.963,6.487]	[3.704,9.553]	[2.576,10.843]	[5.864,13.00]	[3.72,21.909]
CAI (previous 12 months)						
No	Ref	Ref	Ref	Ref	Ref	Ref
Yes	2.826** [1.535,5.203]	1.769*** [1.394,2.245]	1.942*** [1.469,2.566]	2.120** [1.337,3.363]	2.392*** [1.906,3.001]	3.296*** [1.940,5.601]

*OR* odds ratio; *95% CI* 95% confidence intervals in brackets, *CAI* condomless anal intercourse; *SSA* sub-Sahara Africa; *LA&C* Latin America and Caribbean; *MENA* Middle East and North Africa; *SEAR* South-East Asia Region; *EE* Eastern Europe (i.e., former Soviet Union republics including Baltic states); *WPR* Western Pacific Region excluding Australia and New Zealand

*p < 0.05, **p < 0.01, ***p < 0.001

Multivariable analyses for each migrant category (Table 3 with full model parameters) revealed significant associations, and these were found to differ across migrant categories. Nonetheless, age, settlement size, disclosure of homosexual attraction and HIV knowledge were significant predictors of ever testing for HIV in most of the migrant categories.

**Table 3:** Multivariable logistic regression models (aOR and 95% CI) for predicting ever testing for HIV among mMSM

	SSA	LA&C	MENA	SEAR	EE	WPR
	(n = 252)	(n = 1902)	(n = 951)	(n = 398)	(n = 1605)	(n = 340)
Age						
Age	1.050*	1.030***	1.044***	1.022	1.040***	1.012
	[1.006,1.097]	[1.013,1.048]	[1.021,1.067]	[0.988,1.057]	[1.022,1.058]	[0.969,1.058]
Migrant generation status						
1st	Ref	Ref	Ref	Ref	Ref	Ref
2nd/Other	0.497 [0.219,1.130]	0.843 [0.554,1.284]	1.218 [0.845,1.754]	1.210 [0.690,2.122]	1.567 [0.390,6.292]	1.514 [0.560,4.090]
Educational attainment						
Low/Med. Educ	0.716 [0.336,1.525]	0.611** [0.454,0.822]	0.661* [0.466,0.936]	0.752 [0.421,1.345]	0.669** [0.503,0.890]	0.529 [0.260,1.075]
High Educ	Ref	Ref	Ref	Ref	Ref	Ref
Settlement size: size of city/town of residence						
> 500,000	Ref	Ref	Ref	Ref	Ref	Ref
< 500,000	0.446* [0.207,0.963]	0.571*** [0.432,0.755]	0.652** [0.471,0.902]	0.577* [0.334,0.997]	0.672** [0.507,0.890]	0.615 [0.325,1.165]
Sexual identity						
Gay/Homosexual	Ref	Ref	Ref	Ref	Ref	Ref
Not gay/homosexual	0.722 [0.341,1.530]	0.536*** [0.393,0.731]	0.860 [0.593,1.246]	0.910 [0.496,1.670]	0.571*** [0.418,0.781]	1.114 [0.485,2.558]
Occupation						
Employed	Ref	Ref	Ref	Ref	Ref	Ref
Unemployed	0.659 [0.174,2.496]	0.759 [0.457,1.260]	0.589 [0.327,1.061]	2.110 [0.367,12.13]	1.250 [0.630,2.482]	1.265 [0.282,5.672]
Student	0.750 [0.295,1.904]	0.470*** [0.328,0.672]	0.887 [0.575,1.367]	0.548 [0.286,1.051]	0.769 [0.532,1.111]	0.346* [0.150,0.798]
Other	0.404 [0.0896,1.821]	0.856 [0.428,1.714]	1.258 [0.578,2.736]	0.680 [0.151,3.052]	0.713 [0.366,1.389]	0.706 [0.129,3.871]
Financial status/coping						
(Really) comfortable	Ref	Ref	Ref	Ref	Ref	Ref
Neutral	0.937 [0.387,2.267]	0.809 [0.590,1.108]	0.769 [0.525,1.125]	0.828 [0.452,1.514]	0.766 [0.569,1.030]	1.633 [0.729,3.655]
(Really) Struggling	0.900 [0.356,2.280]	1.384 [0.924,2.073]	1.105 [0.702,1.738]	1.134 [0.503,2.556]	0.793 [0.515,1.220]	0.662 [0.292,1.499]
Disclosure of homosexual attraction (outness)						
Out to all/ > half	Ref	Ref	Ref	Ref	Ref	Ref
Out to < half/Few/None	0.952 [0.428,2.120]	0.532*** [0.396,0.715]	0.687* [0.476,0.992]	0.461* [0.255,0.835]	0.541*** [0.404,0.723]	0.630 [0.303,1.310]
HIV testing, treatment and transmission knowledge						
HIV Knowledge	1.454** [1.114,1.897]	1.249*** [1.144,1.363]	1.240*** [1.138,1.352]	1.182 [0.994,1.405]	1.296*** [1.193,1.408]	1.389*** [1.164,1.657]
Number of male sexual partners (previous 12 months)						
0	Ref	Ref	Ref	Ref	Ref	Ref
1–10 partners	2.778 [0.805,9.585]	1.254 [0.786,1.999]	1.292 [0.747,2.235]	1.504 [0.683,3.314]	1.715* [1.130,2.604]	0.774 [0.292,2.053]
> 10 partners	2.346 [0.588,9.367]	2.190** [1.277,3.755]	3.125*** [1.634,5.976]	2.311 [0.872,6.125]	4.395*** [2.578,7.491]	2.551 [0.742,8.770]
CAI (previous 12 months)						
No	Ref	Ref	Ref	Ref	Ref	Ref
Yes	1.791 [0.768,4.180]	1.304 [0.947,1.797]	1.435 [0.994,2.072]	1.434 [0.772,2.666]	1.531** [1.135,2.066]	2.323* [1.131,4.771]
Model *χ*^*2*^(*df*)	34.96(15)**	236.62(15)***	135.23(15)***	43.97(15)***	236.47(15)***	52.67(15)***
Cragg-Uhler/Nagelkerke	0.312	0.260	0.261	0.220	0.293	0.361

*aOR* adjusted odds ratio; *95% CI* 95% confidence intervals in brackets; *CAI* condomless anal intercourse; *SSA* sub-Sahara Africa; *LA&C* Latin America and Caribbean; *MENA* Middle East and North Africa; *SEAR* South-East Asia Region; *EE* Eastern Europe (i.e., former Soviet Union republics including Baltic states); *WPR* Western Pacific Region excluding Australia and New Zealand

*p < 0.05, **p < 0.01, ***p < 0.001

Among mMSM from SSA, we observed decreased odds of ever testing for HIV only in those living in smaller settlements (aOR 0.45, 95%-CI 0.21–0.96, *p* < 0.05). Higher age (aOR 1.05, 95%-CI 1.01–1.10, *p* < 0.05) and those with higher HIV knowledge (aOR 1.45, 95%-CI 1.11–1.90, *p* < 0.01) were more likely to have tested for HIV.

For mMSM from LA&C, ever testing was less likely among those with low/moderate educational attainment (aOR 0.61, 95%-CI 0.45–0.82, *p* < 0.01), living in smaller settlements (aOR 0.57, 95%-CI 0.43–0.76, *p* < 0.001), not self-identifying as gay (aOR 0.54, 95%-CI 0.39–0.73, *p* < 0.001), students (aOR 0.47, 95%-CI 0.33–0.67, *p* < 0.001) and those with limited disclosure of homosexual attraction (aOR 0.53, 95%-CI 0.40–0.72, *p* < 0.001). Higher age (aOR 1.03, 95%-CI 1.01–1.05, *p* < 0.001), those with higher HIV knowledge (aOR 1.25, 95%-CI 1.14–1.36, *p* < 0.001) and more male sex partners (> 10 partners [aOR 2.19, 95%-CI 1.28–3.76, *p* < 0.01]) were more likely to have tested for HIV.

Among mMSM from MENA, ever testing for HIV was less likely in those with low/moderate educational attainment (aOR 0.66, 95%-CI 0.47–0.94, *p* < 0.05), living in smaller settlements (aOR 0.65, 95%-CI 0.47–0.90, *p* < 0.01), and limited disclosure of homosexual attraction (aOR 0.69, 95%-CI 0.48–0.99, *p* < 0.05). Similar to mMSM from LA&C, mMSM from MENA with higher age (aOR 1.04, 95%-CI 1.02–1.07, *p* < 0.001), higher HIV knowledge (aOR 1.24, 95%-CI 1.14–1.35, *p* < 0.001), and more male sex partners (> 10 partners [aOR 3.13, 95%-CI 1.63–5.98, *p* < 0.001]) were also more likely to have ever tested for HIV.

Migrant MSM from SEAR living in smaller settlements (aOR 0.58, 95%-CI 0.33–0.99, *p* < 0.05) and with limited disclosure of homosexual attraction (aOR 0.46, 95%-CI 0.26–0.84, *p* < 0.05) were less likely to have ever tested for HIV.

Among mMSM from EE, ever testing for HIV was less likely among those with low/moderate educational attainment (aOR 0.67, 95%-CI 0.50–0.89, *p* < 0.01), living in smaller settlements (aOR 0.67, 95%-CI 0.51–0.89, *p* < 0.01), not self-identifying as gay (aOR 0.57, 95%-CI 0.42–0.78, *p* < 0.001) and limited disclosure of homosexual attraction (aOR 0.54, 95%-CI 0.40–0.72, *p* < 0.001). On the other hand, higher age (aOR 1.04, 95%-CI 1.02–1.06, *p* < 0.001), higher knowledge of HIV (aOR 1.30, 95%-CI 1.19–1.41, *p* < 0.001), 1–10 male sex partners (aOR 1.72, 95%-CI 1.13–2.60, *p* < 0.05), > 10 male sex partners (aOR 4.40, 95%-CI 2.58–7.49, *p* < 0.001), and those who reported CAI in the previous 12 months (aOR 1.53, 95%-CI 1.13–2.06, *p* < 0.01) had higher odds of ever testing for HIV.

Lastly, mMSM from WPR who were students (aOR 0.35, 95%-CI 0.15–0.80, *p* < 0.05) were less likely to have ever tested for HIV. Higher knowledge of HIV (aOR 1.39, 95%-CI 1.16–1.66, *p* < 0.001) and CAI in the previous 12 months (aOR 2.32, 95%-CI 1.13–4.77, *p* < 0.05) was associated with increased odds of ever testing for HIV.

## Discussion

Using data from the EMIS-2017 survey, we investigated HIV testing prevalence and recency in mMSM residing in 10 European countries, after which we further explored the determinants of HIV testing in mMSM from SSA in comparison to mMSM from other regions included in the study. To the best of our knowledge, no previous study has explored the determinants of HIV testing in mMSM from SSA and the differential impact of these determinants across mMSM categories in these ten European countries.

Our results showed that, overall, one in five (20%) mMSM included in our analysis had never been tested for HIV, a similar proportion as reported in the overall European sample [54]. By contrast, there was a higher proportion of recent testing (previous 12 months) among mMSM in our study compared to the European sample (65% vs. 56%). Furthermore, of those ever tested,15% of mMSM had not tested in the 12 months prior to survey completion. Additionally, our data confirms the determinants of never testing for HIV reported in previous literature among non-migrant MSM [51–53, 61] such as lower age, lower educational attainment, residence in smaller settlements, not identifying as gay, being a student, limited disclosure of homosexual attraction to others, lower HIV knowledge, lower numbers of male sexual partners and less CAI in the previous 12 months. Although our findings are the same as in non-migrant MSM, our analytic approach addresses methodological limitations in past studies regarding the categorization of migration background and provides a more specific assessment of the determinants of HIV testing in the different mMSM categories residing in Europe which can inform the development of interventions to increase testing uptake among mMSM.

We found a higher prevalence of testing in mMSM from SSA than in other mMSM categories except for mMSM from LA&C, and this finding can possibly be explained by the higher perception of risk due to higher prevalence of HIV in SSA (and LA&C) countries. Furthermore, this may be reflective of mMSM from SSA proactively seeking opportunities for testing, in addition to the acceptance of testing when initiated by healthcare providers. Mohammed and colleagues [62], in their study investigating the frequency and correlates of HIV test refusal at sexual health clinics in UK, reported that SSA mMSM were less likely to refuse testing for HIV compared to other SSA heterosexual migrants. Lastly, this finding can also be a result of the cumulative effect of testing and prevention campaigns targeting SSA and MSM communities in Europe. HIV testing prevalence observed in all mMSM categories in this study ranged between 75 and 84% and may be a result of better access to MSM-relevant and competent services. However, this can also arise from a sampling bias in the survey, which attracted more highly educated individuals (68% of our analytic sample were highly educated).

It is worth highlighting that the proportion of those last tested for HIV more than 12 months ago in this study is lower than reported from other studies done in Germany [61] and Australia [63] and could be suggestive of higher recent HIV testing (previous 12 months) among mMSM compared to non-migrant MSM. This is consistent with our finding of higher recent testing in mMSM compared to the overall European sample [54], and a UK study reporting that mMSM were more likely than non-migrant MSM to have received an HIV test in the previous 12 months [29]. Despite these findings, the profiles of untested mMSM in this study who may be at risk for HIV remains a valid source for concern and aligns with literature reporting condomless anal intercourse [64], substance use [23], and low knowledge of testing opportunities for HIV testing [54, 64] in mMSM who have never tested for HIV. A noteworthy finding in our study is the lower testing prevalence among mMSM from MENA, which may be due to religious and cultural norms around sexuality, and which may act as a barrier to accessing HIV testing and prevention services. Notably, 60% of mMSM from MENA countries did not fully disclose their homosexual attraction to others. Our finding of a higher proportion of limited disclosure and lower proportion of HIV testing in this mMSM category reflects findings from past research reporting high levels of never testing for HIV among mMSM from MENA who were not comfortable with disclosing sexual orientation to healthcare providers [64]. Contrary to our expectations, the proportion of limited disclosure was much lower in other mMSM categories (36–53%) and may be partly responsible for the lower proportion of untested mMSM in these categories.

Given our comparative approach to assessing the determinants of HIV testing in mMSM from SSA and comparing with mMSM from other regions, we identified varying salient similarities and differences in the determinants of HIV testing between mMSM from SSA and mMSM from other regions. First, our analysis shows that higher age increased odds of ever testing for HIV in most mMSM categories and is consistent with previous research done within [41–43, 52] and outside Europe [63, 65]. This may be attributed to a lack of accessible services including challenges in designing age-appropriate programs for young (migrant) MSM. While testing opportunities may increase over time in older mMSM compared to younger mMSM, this finding has implications for the current regional epidemiological context. As young MSM disproportionately contribute to new HIV diagnoses in Europe [66], this demands attention. Younger mMSM may not be well equipped in navigating and accessing relevant testing and prevention services [44]. Other barriers may also be related to the non-disclosure of homosexual attraction, perceived stigma, and lack of young or youth MSM friendly services, especially in smaller settlements [44]. Consistent with previous research [41, 42, 52, 53], our analysis also shows that mMSM from LA&C, MENA and EE with lower educational attainment were significantly less likely to have ever tested for HIV. Diaz and colleagues [67] reported that Latin-American and African MSM with lower educational attainment were more likely to be undiagnosed and present late for HIV care. This may suggest a lack of knowledge about HIV, limited information on testing services or facilities, and poor language proficiency/skills [40, 67–70] among individuals with lower educational attainment. Additionally, we also identified those living in smaller settlements (mMSM from SSA, LA&C, MENA, SEAR and EE) as being less likely to have ever tested for HIV. mMSM living in smaller settlements may experience access challenges due to lack of proximity to testing and prevention services [41, 43] including access to related information [71]. In addition, this may also be linked to the lower perception of HIV risk among those living in smaller settlements or rural areas [65, 72]. Lastly, those living in non-urban settings may encounter access barriers based on the stigma related to same-sex sexual behavior because of residence in more homonegative environments as compared to those living in larger/urban settlements where non-heteronormativity may be accepted [72–74]. Our data also shows that mMSM from LA&C, MENA, SEAR and EE shared several other similar significant determinants of HIV testing which were not observed in other mMSM categories. Those not identifying as gay (LA&C and EE) and not fully disclosing their homosexual attraction (LA&C, MENA, SEAR and EE) were less likely to be tested, and this is in line with existing evidence [29, 42, 43, 53, 61]. However, interestingly, sexual identity and disclosure of homosexual attraction was not associated with HIV testing in mMSM from SSA in our univariate and multivariate analyses, and this may be suggestive of testing regardless of sexual identity, attraction and the disclosure of homosexual attraction, possibly as a result of a high perception of HIV risk due to migration from high HIV prevalence settings.

We found that a higher knowledge of HIV significantly increased the odds for HIV testing in all mMSM categories and this further reinforces the relevance of knowledge as a vital component to increasing HIV testing uptake for key and vulnerable populations such as mMSM. Low HIV knowledge can compromise HIV risk perception, enhance risky sexual behavior and limit utilization of HIV testing and prevention services [52, 67, 75]. On the other hand, higher HIV knowledge could also be a result of information acquired during testing. However, the temporal sequence or direction of association between HIV knowledge and testing could not be ascertained due to the cross-sectional design of EMIS. Regardless, it is important to emphasize that knowledge is not the only determinant of HIV testing behavior and intervention developers should not assume that information provision is sufficient to improve HIV testing behavior in mMSM. We also observed that only mMSM from LA&C and WPR who were students were less likely to have ever tested for HIV. Lastly, with regards to sexual behavior, our analysis shows that mMSM from LA&C, MENA and EE with higher numbers of male sexual partners and mMSM from EE and WPR who had CAI in the previous 12 months were more likely to have tested for HIV, thus pointing to the awareness of the risk associated with these behaviors. However, this was not seen in mMSM from SSA, and may be indicative of testing for HIV regardless of sexual behavior. Findings from this study suggest a complex interplay of individual and contextual factors acting as barriers to HIV testing. For most of the mMSM categories, sociodemographic and sexual behavioral factors including HIV knowledge played a role in the likelihood of ever testing for HIV, with the exception of mMSM from SSA and SEAR for whom no significant associations between sexual behavioral factors and ever testing for HIV were found.

### Strength and Limitations

The strengths of this study include the relatively larger subsample of mMSM from SSA and other world regions residing in ten different European countries. To our knowledge, the EMIS survey remains the largest data source of mMSM in Europe. Another strength is our approach to disaggregating mMSM into different categories based on their region of origin and the inclusion of migrant generation status. Our methodological approach to mMSM categorization contributes to the nascent literature on HIV testing among mMSM in Europe and extends our understanding of the determinants of HIV testing and provides new insight into the differences in determinants of HIV testing among mMSM in Europe including potential target profiles for HIV testing and prevention engagement. Lastly, another strength is the use of a multivariable approach and penalized logistic regression method for mMSM categories with relatively small sample sizes to minimize the chances of unreliable and biased estimates.

Findings from this study are nonetheless subject to some limitations. First, we recognize that our approach to the categorization of mMSM into WHO regional groups may obscure certain ethnic characteristics or differences within categories, but this could not be solved due to a number of practical reasons, for example high heterogeneity within categories, potentially leading to small subsample sizes. Nonetheless, our study approach is an improvement on gaps previously highlighted in past studies using different proxies for migrancy in the European region [41–45]. Fine grain (detailed) categorization of mMSM may be more fitting and applicable to country-level or local studies. Second, the cross-sectional design of this study limits our efforts to assessing associations rather than determining causality. Furthermore, given that data were self-reported, there may be recall and social desirability bias present. However, we consider social desirability bias to be relatively minimal because the study was conducted online and anonymously. Third, despite the large total sample size and use of social networks for participant recruitment, this study may not be representative of mMSM in the selected countries. Participants included in our study sample were mostly highly educated, employed, and comfortable financially. Migrant MSM with low social media and internet presence or use, and those experiencing severe economic hardship may not have participated in the EMIS-2017 survey. Lastly, for our analysis, we did not include individual country of residence in our logistic regression models because of small subsample sizes for some migrant categories. Hence, our analysis is based solely on a shared characteristics perspective. It is important to highlight that ethnic-racial disparities can differ from one geographical region or country to the other [76]. Similarly, we did not include the length of time (years) of residence in the county in our analysis due to small subsample sizes, as previously mentioned. However, it is important to highlight that this may have implications for the levels of integration, acculturation, HIV-related knowledge, awareness of testing opportunities, access to services, and health-seeking behavior among mMSM [6, 40, 46]. Despite these limitations, we believe that this study can be of benefit in further developing research studies looking to conduct in-depth assessment of the determinants and mechanisms underlying HIV testing in the different categories of mMSM.

### Recommendations for Practice and Future Research

Based on our findings, we recommend three strategies to enhance HIV testing uptake among mMSM in Europe. First, HIV testing and prevention efforts should target the following mMSM subgroups: young mMSM, mMSM residing in smaller settlements, mMSM with lower educational attainment, and mMSM who are students. Second, there is a critical need for tailored HIV education and awareness in mMSM target profiles previously outlined, and this may require the use of cultural and linguistically competent strategies such as bilingual/bicultural staff or cultural mediators in providing information [77] and use of culturally appropriate communication tools like comic books, proverbs, theater and drama in multiple languages [78]. Clearly, proactive and meaningful engagement, and shared decision-making with mMSM should be central to the development of relevant HIV testing and prevention tools, strategies, and interventions. Third, we recommend that HIV service providers consider different tailored approaches to increase the accessibility and acceptability of services offered to mMSM. For this to yield the needed results and impact, these services need to be low-threshold, non-judgmental, MSM-friendly, and culturally appropriate for the different categories of mMSM. Furthermore, there is a need for healthcare providers to create opportunities for facilitating and supporting the disclosure of same-sex attraction in mMSM to enhance access to relevant testing and prevention services.

Our findings also provide avenues for future research. First, in-depth (qualitative) studies investigating the barriers and facilitators of HIV testing among the different mMSM categories are needed to better understand factors influencing HIV testing. This can further contextualize the determinants of HIV testing for each individual local context. Second, additional studies investigating factors influencing HIV testing among mMSM living in small cities, towns and non-urban areas should be conducted as they can inform upcoming national strategic planning processes and ensure effective health resource allocation. Third, HIV testing recency and patterns (history) across the different mMSM categories in different settings should be taken into consideration in future studies as this can further enable the identification of which mMSM should be prioritized for HIV testing and prevention interventions. This should also involve the identification of specific HIV testing and prevention needs, including best approaches for reaching key mMSM subgroups. Lastly, there is a need for more community-driven research including improvement in data collection, especially among mMSM from SSA. Further improvement of the approach used in our study to disaggregate mMSM categories in country-level studies is required, as local contexts may differ from country to country.

## Conclusion

We sought to assess HIV testing prevalence and recency and explore the differences in the determinants of HIV testing in mMSM from SSA and mMSM from other regions. Our data shows a higher prevalence of HIV testing in mMSM from SSA than in other mMSM categories (except for mMSM from LA&C), with a significant proportion of mMSM still untested for HIV who may potentially engage in sexual behavior that puts them at risk of transmitting or acquiring HIV. Furthermore, compared to mMSM from other regions, our analyses suggests that MSM from SSA test for HIV regardless of sexual identity and behavior, and the disclosure of homosexual attraction. Taken together, our analysis provides evidence that, overall, the determinants of ever testing for HIV are largely the same as reported in non-migrant MSM. Nonetheless, these determinants vary across the different migrant categories which suggests that a “one size fits all” approach to HIV testing and prevention for all mMSM is unlikely to improve HIV testing uptake. Furthermore, we identified important sociodemographic and behavioral determinants for targeted HIV testing and prevention interventions among mMSM. Increasing HIV testing uptake among never-tested mMSM remains key to attaining the 95–95-95 UNAIDS targets in European countries yet to do so. It is imperative that mMSM are not left behind in national, regional and global HIV response efforts.

## Data Availability

Available on request to coordinator@emis-project.eu.

## Annex

See Tables 4, 5, 6, 7

**Table 4:** Migrant MSM from SSA by country of residence prior to migration background categorization

Country of residence	SSA
Belgium	28
France	113
Germany	58
Greece	4
Italy	18
Netherlands	25
Portugal	85
Spain	16
Switzerland	11
United Kingdom	215
Total (N)	573

*SSA* sub-Sahara Africa

**Table 5:** Participant (mMSM) country of origin

Region	Participant country of origin
SSA	Angola, Belgium, Benin, Botswana, Burkina Faso, Burundi, Cameroon Cape Verde, Central African Republic, Chad, Comoros, Congo—Kinshasa Congo—Brazzaville, Equatorial Guinea, Eritrea, Ethiopia, Gambia, Ghana, Guinea, Guinea-Bissau, Ivory Coast, Kenya, Liberia, Madagascar, Mali, Mauritania, Mauritius, Mozambique, Namibia, Nigeria, Rwanda, Senegal, Sierra Leone, South Africa, Tanzania, Togo, Uganda, Zambia, Zimbabwe
LA&C	Antigua and Barbuda, Argentina, Bahamas, Barbados, Bolivia, Brazil, Chile, Colombia, Costa Rica, Cuba, Dominica, Dominican Republic, Ecuador, El Salvador, France Overseas Departments, Territories and Collectivities, Guatemala, Guyana, Haiti, Honduras, Jamaica, Mexico, Nicaragua, Panama, Paraguay, Peru, Puerto Rico, Saint Lucia, Suriname, Trinidad and Tobago, Uruguay, Venezuela
MENA	Afghanistan, Algeria, Bahrain, Egypt, Iran, Iraq, Israel, Jordan, Kuwait, Lebanon, Libya, Morocco, Oman, Pakistan, Palestine, Qatar, Saudi Arabia, Syria, Tunisia, Turkey, United Arab Emirates, Yemen
SEAR	Bangladesh, India, Indonesia, Malaysia, Maldives, Singapore, Sri Lanka, Thailand, Timor-Leste
EE	Armenia, Azerbaijan, Belarus, Bulgaria, Croatia, Czech Republic, Estonia, Georgia, Hungary, Kazakhstan, Kyrgyzstan, Latvia, Lithuania, Moldova, Poland, Romania, Russia (Russian Federation), Slovakia, Slovenia, Tajikistan, Turkmenistan, Ukraine, Uzbekistan
WPR	Brunei, Cambodia, China, Indonesia, Japan, South Korea, Laos, Malaysia, Mongolia, Philippines, Singapore, Solomon Islands, Taiwan, Vanuatu, Vietnam

*SSA* sub-Sahara Africa; *LA&C* Latin America and Caribbean; *MENA* Middle East and North Africa; *SEAR* South-East Asia Region; *EE* Eastern Europe (i.e., former Soviet Union republics including Baltic states); *WPR* Western Pacific Region excluding Australia and New Zealand

**Table 6:** All migrant MSM (1st and 2nd/other generation) by country of residence across the 10 countries included in the analysis, and by WHO region of origin

Country of residence	SSA	LA&C	MENA	SEAR	EE	WPR	Total (N)
Belgium	14	55	58	12	71	13	223
France	83	184	290	57	87	55	756
Germany	33	231	387	90	669	67	1477
Greece	2	6	31	1	48	0	88
Italy	15	171	74	14	125	29	428
Netherlands	17	80	63	64	99	22	345
Portugal	46	145	3	5	19	2	220
Spain	9	1051	43	9	140	17	1269
Switzerland	7	69	31	19	72	13	211
United Kingdom	85	189	145	187	542	157	1305
Total (N)	311	2181	1125	458	1872	375	6322

*SSA* sub-Sahara Africa; *LA&C* Latin America and Caribbean; *MENA* Middle East and North Africa; *SEAR* South-East Asia Region; EE, Eastern Europe (i.e., former Soviet Union republics including Baltic states); *WPR* Western Pacific Region excluding Australia and New Zealand

**Table 7:** Multivariable logistic regression models (aOR and 95% CI) for predicting ever testing for HIV among mMSM excluding participants with discrepant data

	SSA	LA&C	MENA	SEAR	EE	WPR
	(n = 214)	(n = 1621)	(n = 801)	(n = 362)	(n = 1420)	(n = 294)
Age						
Age	1.051* [1.000,1.104]	1.036*** [1.017,1.054]	1.049*** [1.023,1.075]	1.023 [0.988,1.060]	1.038*** [1.020,1.057]	0.994 [0.950,1.040]
Migrant generation status						
1st	Ref	Ref	Ref	Ref	Ref	Ref
2nd/Other	0.461 [0.185,1.153]	0.846 [0.544,1.317]	1.070 [0.724,1.581]	1.143 [0.638,2.049]	1.344 [0.325,5.553]	1.511 [0.550,4.147]
Educational attainment						
Low/Med. Educ	0.726 [0.318,1.654]	0.583*** [0.424,0.803]	0.723 [0.493,1.061]	0.708 [0.389,1.291]	0.703* [0.520,0.951]	0.553 [0.258,1.184]
High Educ	Ref	Ref	Ref	Ref	Ref	Ref
Settlement size: size of city/town of residence						
> 500,000	Ref	Ref	Ref	Ref	Ref	Ref
< 500,000	0.483 [0.206,1.131]	0.557*** [0.413,0.751]	0.639* [0.448,0.913]	0.545* [0.308,0.962]	0.624** [0.463,0.843]	0.597 [0.302,1.182]
Sexual identity						
Gay/Homosexual	Ref	Ref	Ref	Ref	Ref	Ref
Not gay/homosexual	0.813 [0.353,1.874]	0.541*** [0.387,0.757]	0.816 [0.541,1.230]	0.921 [0.490,1.730]	0.529*** [0.378,0.740]	0.962 [0.396,2.333]
Occupation						
Employed	Ref	Ref	Ref	Ref	Ref	Ref
Unemployed	0.764 [0.177,3.298]	0.772 [0.452,1.319]	0.650 [0.337,1.253]	1.838 [0.314,10.74]	1.220 [0.590,2.523]	0.947 [0.216,4.155]
Student	0.799 [0.283,2.251]	0.465*** [0.317,0.682]	0.965 [0.602,1.548]	0.597 [0.303,1.177]	0.672* [0.456,0.991]	0.221** [0.0875,0.558]
Other	0.373 [0.0678,2.046]	0.708 [0.335,1.497]	1.080 [0.473,2.466]	0.657 [0.144,2.999]	0.653 [0.326,1.308]	0.740 [0.133,4.108]
Financial status/coping						
(Really) comfortable	Ref	Ref	Ref	Ref	Ref	Ref
Neutral	0.755 [0.282,2.023]	0.860 [0.614,1.204]	0.709 [0.469,1.074]	0.865 [0.463,1.617]	0.788 [0.575,1.080]	1.894 [0.799,4.491]
(Really) Struggling	0.878 [0.309,2.497]	1.541 [0.996,2.384]	1.094 [0.667,1.797]	1.474 [0.605,3.590]	0.895 [0.561,1.427]	0.653 [0.275,1.554]
Disclosure of homosexual attraction (outness)						
Out to all/ > half	Ref	Ref	Ref	Ref	Ref	Ref
Out to < half/Few/None	0.836 [0.351,1.988]	0.527*** [0.384,0.724]	0.591** [0.397,0.879]	0.437** [0.235,0.812]	0.592*** [0.436,0.806]	0.579 [0.265,1.265]
HIV testing, treatment and transmission knowledge						
HIV Knowledge	1.379* [1.008,1.888]	1.250*** [1.135,1.376]	1.275*** [1.157,1.406]	1.189 [0.992,1.423]	1.329*** [1.210,1.459]	1.346** [1.115,1.624]
Number of male sexual partners (previous 12 months)						
0	Ref	Ref	Ref	Ref	Ref	Ref
1–10 partners	2.765 [0.769,9.936]	1.132 [0.701,1.829]	1.257 [0.711,2.222]	1.559 [0.692,3.509]	1.730* [1.130,2.647]	0.744 [0.268,2.063]
> 10 partners	2.273 [0.536,9.640]	2.169** [1.229,3.829]	3.273*** [1.625,6.594]	2.463 [0.904,6.712]	4.711*** [2.686,8.261]	2.202 [0.619,7.838]
CAI (previous 12 months)						
No	Ref	Ref	Ref	Ref	Ref	Ref
Yes	2.074 [0.816,5.269]	1.261 [0.897,1.772]	1.354 [0.908,2.020]	1.484 [0.777,2.834]	1.526** [1.111,2.098]	2.060 [0.958,4.429]
Model *χ*^*2*^(*df*) 28.07(15)*	28.07(15)*	218.58(15)***	124.03(15)***	42.50(15)***	218.87(15)***	45.95(15)***
Cragg-Uhler/Nagelkerke	0.314	0.282	0.292	0.234	0.309	0.367

*aOR* adjusted odds ratio; *95% CI* 95% confidence intervals in brackets; *CAI* condomless anal intercourse; *SSA* sub-Sahara Africa; *LA&C* Latin America and Caribbean; *MENA* Middle East and North Africa; *SEAR* South-East Asia Region; *EE* Eastern Europe (i.e., former Soviet Union republics including Baltic states); *WPR* Western Pacific Region excluding Australia and New Zealand

**p* < 0.05, ***p* < 0.01, ****p* < 0.001
